# Diet quality of children in the United States by body mass index and sociodemographic characteristics

**DOI:** 10.1002/osp4.388

**Published:** 2019-12-17

**Authors:** Jessica L. Thomson, Alicia S. Landry, Lisa M. Tussing‐Humphreys, Melissa H. Goodman

**Affiliations:** ^1^ US Department of Agriculture Agricultural Research Service Stoneville Mississippi; ^2^ Department of Family and Consumer Sciences University of Central Arkansas Conway Arkansas; ^3^ Department of Medicine and Cancer Center University of Illinois at Chicago Chicago Illinois

**Keywords:** body weight status, child age, Healthy Eating Index, race/ethnicity

## Abstract

**Objective:**

The primary objective was to use the Healthy Eating Index‐2015 (HEI‐2015) to describe diet quality by categories of body mass index (BMI) and by sociodemographic characteristics within categories of BMI using a nationally representative sample of US children.

**Methods:**

Dietary datasets from three cycles of the National Health and Nutrition Examination Survey (2009‐2014) were analysed for children 2 to 18 years of age (N = 8894). Using the population ratio method, mean and 95% confidence intervals for HEI‐2015 total and component scores were computed by BMI (underweight, normal weight, overweight, and obese) and by age (2‐5, 6‐11, and 12‐18 y), gender, race/ethnicity (non‐Hispanic black, non‐Hispanic white, Mexican American, other Hispanic, and other race), and family poverty to income ratio (below and at/above poverty threshold).

**Results:**

HEI‐2015 mean total scores were 50.4, 55.2, 55.1, and 54.0 out of 100 points for children with underweight, normal weight, overweight, and obesity, respectively, and were not significantly different. Within BMI categories, significant differences in total and mean component scores were present for age and race/ethnicity groups.

**Conclusions:**

Total and most components of diet quality did not significantly differ among child populations classified by BMI status. Within BMI categories, significant diet quality differences were found for age and race/ethnicity groups, although scores were low for all child groups. Researchers may need to address or target specific dietary components with low quality in various child populations to have the greatest effect on improving nutrition nationwide.

## 1 INTRODUCTION

Assessment of diet quality involves both the quality and variety of a holistic diet, rather than individual nutrients, and can evaluate how closely eating patterns align with dietary recommendations 1. A recent systematic review and meta‐analysis of prospective cohort studies found that diets scoring highly on several diet quality indices were associated with significant reductions in risk of all‐cause mortality, cardiovascular disease, cancer, and type 2 diabetes in adults 2. Because unhealthy dietary behaviours established early in life can lead to obesity in childhood that follows into adulthood 3, assessing the diet quality of children is important for addressing current and future health issues. Determining diet quality among child populations defined by weight status is crucial given the high prevalence of childhood obesity present in the United States. The relationship between diet quality and weight status in children has been studied by others, but results are inconclusive because both negative and positive associations have been reported 1, 4. Several sociodemographic factors have been associated with diet quality in children, including socio‐economic status (positive association), age (negative association), sex (higher for girls), race/ethnicity (lower for black children, higher for Mexican American children), and geographic location (lower for rural) 1, 5, but their interactions with weight status have not been extensively studied. Further exploration of the relationships among diet quality, weight status, and sociodemographic characteristics of children is needed as such information may be important for understanding obesity disparities in child populations and can aid in designing interventions to improve diet quality in children, especially those targeting specific populations.

The Healthy Eating Index (HEI) is a reliable and validated diet quality index that measures how well individuals in the United States aged 2 years and older meet dietary recommendations promoted by the Dietary Guidelines for Americans 6. Because scoring for the HEI operates on a density basis (eg, amount per 1000 kcal, ratio of fatty acids), diet quality is assessed independent of quantity 7. The current version, HEI‐2015, corresponds to the 2015‐2020 Dietary Guidelines for Americans, and higher index scores have been associated with reduced risk for cardiovascular disease, cardiovascular disease mortality, and all‐cause mortality in a prospective cohort of black and white adults 8. HEI‐2015 scores can be interpreted both quantitatively (score range = 0‐100) and qualitatively (10‐point grading scale; A = 90‐100 points, B = 80‐89 points, C = 70‐79, D = 60‐69 points; F = 0‐59 points) to describe adherence to the Guidelines 9. For the US population overall and by child and adult status, HEI‐2015 scores are available on the US Department of Agriculture's Center for Nutrition Policy and Promotion website 10. However, to date, HEI‐2015 scores have not been published in the peer‐reviewed literature for nationally representative samples of children in the United States by body mass index (BMI) category. The purpose of this research was to use the HEI‐2015 to describe and provide reliable estimates of diet quality of children in the United States by BMI category and by sociodemographic characteristics (age, gender, race/ethnicity, and poverty status) within BMI category. These analyses provide estimates of nationally representative total diet quality and component scores within BMI categories that can be employed by researchers to compare child populations of interest and to identify dietary components that score especially low for quality.

## 2 METHODS

### 2.1 Study design and data source

The National Health and Nutrition Examination Survey (NHANES) is a programme of studies designed to assess the health and nutritional status of noninstitutionalized children and adults in the United States 11. The NHANES interview includes demographic, socio‐economic, dietary, and health‐related questions. The 2009 to 2010, 2011 to 2012, and 2013 to 2014 cycles of NHANES data were used for this analysis because they were the most recent cycles with available dietary data at the time the analyses were conducted. To obtain reliable estimates for child populations, three cycles of data were used. The NHANES protocol was approved by the National Center for Health Statistics Research Ethics Review Board (protocol #2005‐06 and #2011‐17). Informed consent was obtained from participants 18 years of age and older. Written parental consent and child assent was obtained from participants 2 to 17 years of age.

### 2.2 Dietary intake

Two days of dietary intake were collected using the US Department of Agriculture (USDA) Automated Multiple‐Pass Method (AMPM), a fully computerized instrument for collecting 24‐hour dietary recalls either in person or by telephone 12. The first day's interview was conducted in person in the Mobile Examination Center. The second day's interview was collected by telephone 3 to 10 days after the first day. Only the first day's data were used in these analyses. Proxies reported dietary intake for children 5 years of age and younger and assisted with the dietary interview for children 6 to 11 years of age. Children 12 years of age and older self‐reported dietary intake. Only recalls that were coded as reliable and met the minimum criteria (first four steps of five‐step AMPM were completed and food/beverages consumed for each reported eating occasion identified) were included in the analyses. Recalls were checked for consumption of human milk, which can be problematic because amounts are not quantified causing missing values for amounts of energy and nutrients from human milk. No child reported consuming human milk on the first day of intake; hence, no recalls were excluded for this reason. Of the 9000 participants between 2 and 18 years of age with reliable dietary recalls, 106 were excluded due to missing BMI data.

### 2.3 HEI‐2015 scores

To compute HEI‐2015 scores, nutrient content and food serving equivalents are needed. We used the USDA Food and Nutrient Database for Dietary Studies, versions 5, 2011‐2012, and 2013‐2014 13, to code nutrient content of the dietary intake data. We used the USDA Food Patterns Equivalent Database, versions 2009‐2010, 2011‐2012, and 2013‐2014 14, to compute food serving equivalents. Total HEI‐2015 scores can range from 0 to 100 and are computed by summing scores for 13 components. The nine adequacy components are as follows: total fruits, whole fruits, total vegetables, greens and beans, whole grains, dairy, total protein foods, seafood and plant proteins, and fatty acids. The four moderation components are as follows: refined grains, sodium, added sugars, and saturated fats. Higher intakes of adequacy components result in higher scores (increase intake for healthier diet) and maximum scores are based upon meeting or exceeding the lowest recommended amount for each component. Lower intakes of moderation components result in higher scores (decrease intake for healthier diet), and maximum scores are based upon meeting or remaining under the recommended limit for each component. Additional details of the HEI‐2015 scoring standards can be found on the National Cancer Institute's website 15.

### 2.4 BMI and sociodemographic characteristics

Body measurements were conducted in the Mobile Examination Center by trained health technicians. BMI was calculated as weight (kg) divided by height (m^2^), and categories were based on the Centers for Disease Control and Prevention's sex‐specific 2000 BMI‐for‐age growth charts. The four categories are underweight (BMI < 5th percentile), normal weight (5th percentile ≤ BMI < 85th percentile), overweight (85th percentile ≤ BMI < 95th percentile), and obese (≥95th percentile). Age, gender, race/ethnicity, and family income were self‐reported directly by participants 16 years of age or older and emancipated minors. For participants less than 16 years of age or those who could not answer the questions themselves, a proxy (eg, parent or guardian) provided this information. For analytic purposes, we categorized age into three groups—2 to 5 years, 6 to 11 years, and 12 to 18 years—based on mode of dietary recall (proxy, proxy assistance, and self‐report) and education level (preschool, grade school, and middle/high school). Race/ethnicity included five categories—non‐Hispanic black, non‐Hispanic white, Mexican American, other Hispanic, and other race (including multiracial). Poverty to income ratio (PIR) was calculated by dividing family income by the Department of Health and Human Services poverty guidelines specific to the survey year. The PIR value was not computed if income was missing or insufficiently reported for computational purposes (ie, in dichotomous form as <$20 000 or ≥$20 000). PIR values at or above 5.00 were coded as ≥5.00 because of disclosure concerns (eg, potential for participant identification). For analytic purposes, we dichotomized PIR as <1.00 (below poverty threshold) or ≥ 1.00 (at or above poverty threshold). For succinctness, children living in families with income below the poverty threshold were identified as PIR < 1 and children living in families with income at or above the poverty threshold were identified as PIR ≥ 1.

### 2.5 Statistical analyses

Statistical analyses were performed using SAS software, version 9.4 (SAS Institute Inc, Cary, North Carolina). BMI and sociodemographic characteristics of the child dataset were summarized using procedures designed for complex sample surveys that can incorporate cluster, strata, and weight variables. Six‐year weights were constructed for these analyses since three cycles of NHANES data were used. Based on SAS programs and examples provided by the National Cancer Institute 15, the population ratio method was used to estimate intakes of dietary components for use in calculating HEI‐2015 scores. For this method, intake of the relevant dietary constituents and energy were summed for all children in the population of interest to obtain estimates of the population's total intake, and then ratios of the constituent to energy were computed and scored 15. Usual intake at the population level is estimated using the population ratio method. To aid in visualization of the multidimensional qualities of HEI‐2015, the 13 component scores were graphed using radar charts. Component scores were converted to percentages of the maximum score such that the outer edge of the circle (or spoke) represented a score that is 100% of the maximum score for that component, while the centre of the circle represents a score of 0% for any component. Additionally, HEI‐2015 mean total scores and 95% confidence intervals were graphed using interval plots. Because no direct test exists for making statistical comparisons between groups when using the population ratio method 15, comparisons were based upon the computed 95% confidence intervals. If the confidence intervals overlapped between two child populations, it was concluded that a significant difference did not exist. Conversely, if confidence intervals did not overlap between two populations, it was concluded that a significant difference did exist. Due to small sample sizes, estimates were unreliable for race/ethnicity within the underweight category, and therefore, these estimates were not reported.

## 3 RESULTS

Anthropometric and sociodemographic characteristics of the children included in the analytic dataset (N = 8894) are presented in Table 1. The majority (64%) of children were classified with normal weight while one‐third (33%) were classified with overweight or obesity. Percentages of children increased with ascending age group. Percentages of boys and girls in the analytic sample were approximately equal. The majority of children were non‐Hispanic white (55%) with other Hispanic representing the smallest racial/ethnic group (7%). Approximately three‐fourths of the children lived in families that were at or above the poverty threshold.

**Table 1:** Anthropometric and sociodemographic characteristics for children 2‐18 years of age: NHANES 2009‐2014 (N = 8894)

Characteristic	n^a^	%^b^	95% CI	
BMI^c^, kg/m^2^				
Underweight	305	3.3	2.7	3.8
Normal weight	5590	64.2	62.6	65.7
Overweight	1396	15.5	14.5	16.6
Obese	1603	17.0	15.9	18.2
Age, y				
2‐5	2310	22.3	21.2	23.4
6‐11	3334	33.9	32.6	35.2
12‐18	3250	43.8	42.2	45.4
Gender				
Male	4538	50.8	49.3	52.3
Female	4356	49.2	47.7	50.7
Ethnicity				
Non‐Hispanic black	2190	13.9	11.5	16.3
Non‐Hispanic white	2456	54.7	49.6	59.8
Mexican American	2083	15.4	12.0	18.8
Other Hispanic	991	7.3	5.5	9.1
Other race^d^	1174	8.8	7.5	10.0
Poverty income ratio				
Below (<1)	2797	23.9	21.1	26.8
At or above (≥1)	5349	76.1	73.2	78.9

Abbreviations: BMI, body mass index; CI, confidence interval; NHANES, National Health and Nutrition Examination Survey.

a Sample sizes are unweighted.

b Percentages & CI adjusted for 6‐year survey weights.

c Underweight BMI < 5th percentile; normal weight 5th ≤ BMI < 85th percentile; overweight 85th ≤ BMI < 95th percentile; obese BMI ≥ 95th percentile.

d Includes multiracial.

### 3.1 HEI‐2015 total scores

**Figure 1:**
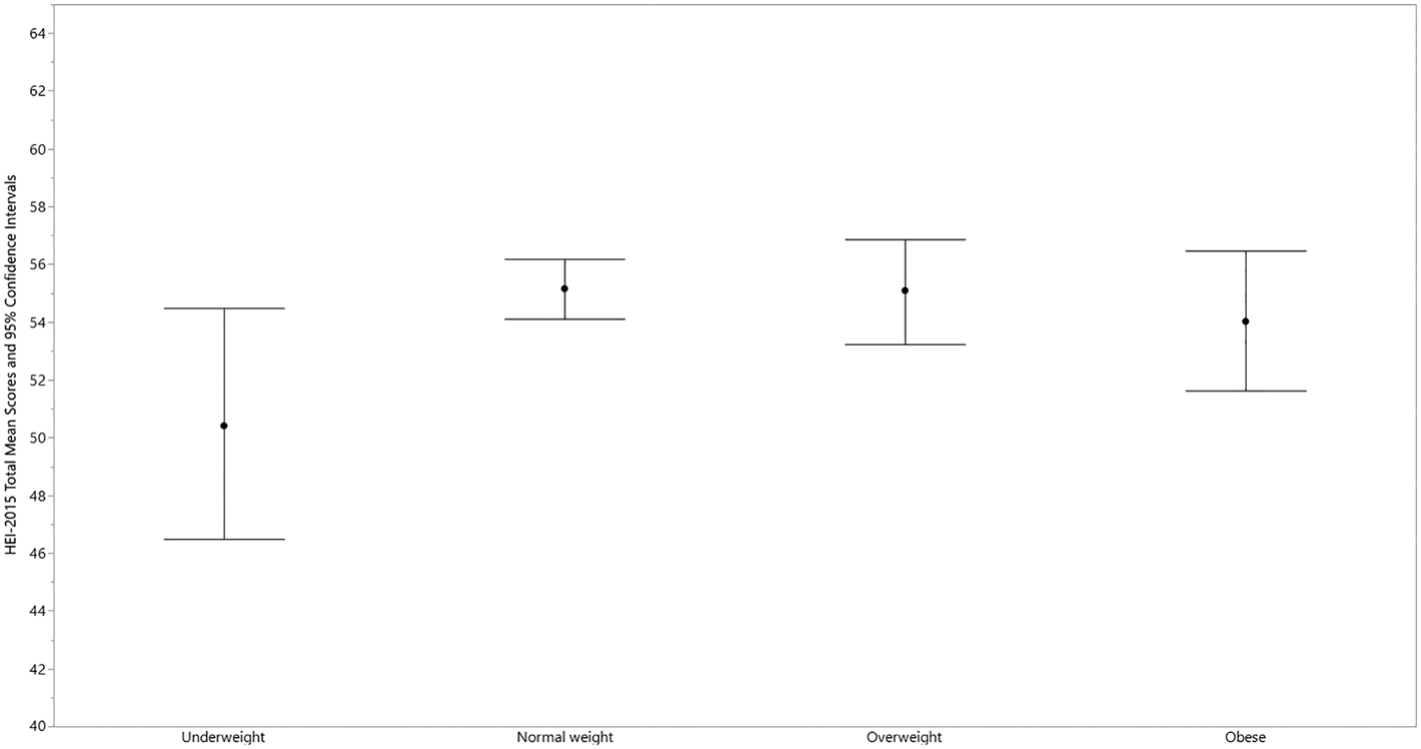
HEI‐2015 total mean scores and 95% confidence intervals for children 2‐18 years of age by body mass index category: NHANES 2009‐2014. HEI scores are calculated using population ratio method. Body mass index (BMI) categories: underweight BMI < 5th percentile; normal weight 5th ≤ BMI < 85th percentile; overweight 85th ≤ BMI < 95th percentile; obese BMI ≥ 95th percentile. HEI, Healthy Eating Index; NHANES, National Health and Nutrition Examination Survey

**Figure 2:**
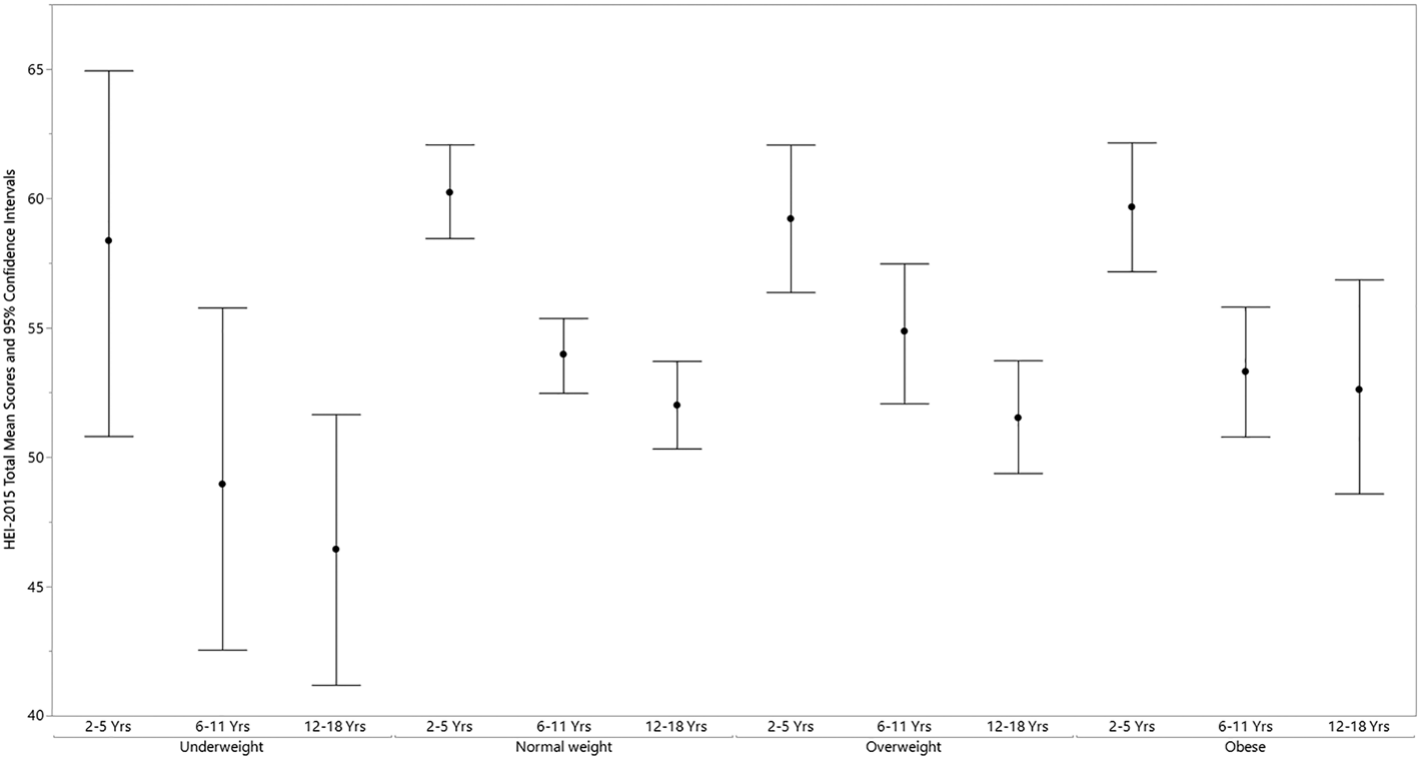
HEI‐2015 total mean scores and 95% confidence intervals for children 2‐18 years of age by age group within body mass index category: NHANES 2009‐2014. HEI scores are calculated using population ratio method. Body mass index (BMI) categories: underweight BMI < 5th percentile; normal weight 5th ≤ BMI < 85th percentile; overweight 85th ≤ BMI < 95th percentile; obese BMI ≥ 95th percentile. HEI, Healthy Eating Index; NHANES, National Health and Nutrition Examination Survey; Yrs, years

**Figure 3:**
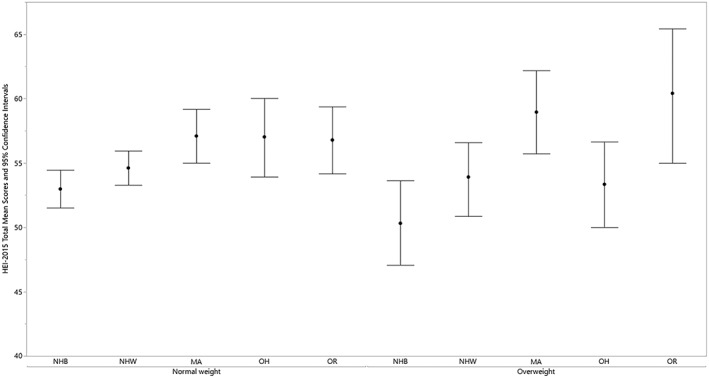
HEI‐2015 total mean scores and 95% confidence intervals for children 2‐18 years of age by race/ethnicity within body mass index category: NHANES 2009‐2014. HEI scores are calculated using population ratio method. Body mass index (BMI) categories: normal weight 5th ≤ BMI < 85th percentile; overweight 85th ≤ BMI < 95th percentile; underweight and obese categories not shown due to unreliable estimates and no significant differences, respectively. HEI, Healthy Eating Index; NHANES, National Health and Nutrition Examination Survey; NHB, non‐Hispanic black; NHW, non‐Hispanic white; MA, Mexican American, OH, other Hispanic; OR, other race

HEI‐2015 mean total scores for children by BMI category and by age, gender, race/ethnicity, and PIR within BMI category are presented in Tables 2, 3, 4, 5, 6 and Figures 1, 2, 3. Mean total scores were 50.4, 55.2, 55.1, and 54.0 for children with underweight, normal weight, overweight, and obesity, respectively, and were not significantly different (Table 2 and Figure 1). Significant differences were present for age group within BMI category (Table 3 and Figure 2). Mean total scores were significantly higher for the youngest versus the two older age groups for children with normal weight (60.2 vs 54.0 and 52.0) and for children with obesity (59.7 vs 53.3 and 52.6). Mean total scores were significantly higher for the youngest versus the oldest age group for children with overweight (59.2 vs 51.5).

**Table 2:** HEI‐2015 scores for children 2‐18 years of age by BMI category: NHANES 2009‐2014 (N = 8894)

		Underweight (n = 305)			Normal Weight (n = 5590)			Overweight (n = 1396)			Obese (n = 1603)		
Component	Range	Mean	95% CI		Mean	95% CI		Mean	95% CI		Mean	95% CI	
Total fruits	0‐5	3.1	2.6	3.7	3.8	3.6	4.0	3.7	3.3	4.1	3.3	3.0	3.7
Whole fruits	0‐5	3.8	3.1	4.5	4.7	4.4	5.0	4.6	3.9	5.0	4.2	3.7	4.7
Total vegetables	0‐5	2.0	1.7	2.3	2.3	2.2	2.3	2.3	2.2	2.4	2.5	2.2	2.7
Greens & beans	0‐5	1.5	0.7	2.4	1.6	1.4	1.8	1.7	1.4	2.0	1.9	1.2	2.5
Whole grains	0‐10	2.4	1.8	2.9	2.7	2.5	2.9	2.4	2.1	2.7	2.3	2.0	2.5
Dairy	0‐10	9.5	8.3	10.0	9.0	8.7	9.3	8.7	8.3	9.0	8.6	8.2	9.1
Total protein foods	0‐5	4.3	3.9	4.7	4.5	4.3	4.7	4.9	4.5	5.0	5.0	4.8	5.0
Seafood & plant proteins	0‐5	2.8	2.0	3.6	3.0	2.7	3.4	3.1	2.7	3.5	2.9	2.5	3.4
Fatty acids	0‐10	1.9	1.0	2.8	3.1	2.8	3.4	3.3	2.9	3.7	3.6	3.1	4.0
Refined grains	0‐10	4.4	3.4	5.3	4.7	4.5	5.0	4.9	4.4	5.4	4.6	4.2	5.1
Sodium	0‐10	4.8	4.0	5.6	4.7	4.5	5.0	4.5	4.1	4.9	3.9	3.5	4.4
Added sugars	0‐10	5.8	4.9	6.6	5.5	5.3	5.8	5.7	5.3	6.0	5.5	5.1	5.9
Saturated fats	0‐10	4.2	3.2	5.2	5.6	5.4	5.8	5.4	4.9	5.8	5.8	5.4	6.2
Total score	0‐100	50.4	46.5	54.5	55.2	54.1	56.2	55.1	53.2	56.9	54.0	51.6	56.5

*Note.* HEI scores are calculated using population ratio method. Underweight BMI < 5th percentile; normal weight 5th ≤ BMI < 85th percentile; overweight 85th ≤ BMI < 95th percentile; obese BMI ≥ 95th percentile.

Abbreviations: BMI, body mass index (kg/m^2^); CI, confidence interval; HEI, Healthy Eating Index; NHANES, National Health and Nutrition Examination Survey.

**Table 3:** HEI‐2015 scores for children 2‐18 years of age by BMI category and age group: NHANES 2009‐2014 (N = 8894)

		Underweight (BMI < 5th Percentile)								
		2‐5 y (n = 102)			6‐11 y (n = 113)			12‐18 y (n = 90)		
Component	Range	Mean	95% CI		Mean	95% CI		Mean	95% CI	
Total fruits	0‐5	4.8	3.8	5.0	3.1	2.2	4.1	2.0	1.4	2.6
Whole fruits	0‐5	4.9	4.3	5.0	3.7	2.6	4.8	2.8	1.9	3.6
Total vegetables	0‐5	2.0	1.4	2.6	1.8	1.3	2.3	2.3	1.8	2.8
Greens & beans	0‐5	2.0	0.7	3.3	1.1	0.3	1.9	1.9	0.0	3.8
Whole grains	0‐10	2.6	1.9	3.2	2.5	1.4	3.7	2.1	1.1	3.2
Dairy	0‐10	9.9	9.1	10.0	9.5	7.5	10.0	8.5	6.7	10.0
Total protein foods	0‐5	4.0	3.1	4.9	3.6	2.9	4.5	5.0	4.6	5.0
Seafood & plant proteins	0‐5	3.8	1.9	5.0	3.0	1.8	4.2	2.1	0.8	3.3
Fatty acids	0‐10	2.0	1.0	3.2	1.8	0.3	3.7	1.9	0.9	3.0
Refined grains	0‐10	5.0	2.9	7.1	4.0	2.5	5.4	4.6	2.7	6.5
Sodium	0‐10	5.8	4.6	7.0	5.4	4.3	6.4	3.6	2.0	5.3
Added sugars	0‐10	6.5	5.6	7.3	5.5	4.2	6.8	5.8	4.6	6.9
Saturated fats	0‐10	5.1	4.0	6.1	4.1	2.5	5.8	3.9	2.6	5.2
Total score	0‐100	58.4	50.8	64.9	49.0	42.6	55.8	46.4	41.2	51.7
		Normal Weight (5th ≤ BMI < 85th Percentile)								
		2‐5 y (n = 1619)			6‐11 y (n = 2024)			12‐18 y (n = 1947)		
Component	Range	Mean	95% CI		Mean	95% CI		Mean	95% CI	
Total fruits	0‐5	5.0	5.0	5.0	3.8	3.5	4.1	2.8	2.6	3.1
Whole fruits	0‐5	5.0	5.0	5.0	4.9	4.6	5.0	3.6	3.1	4.0
Total vegetables	0‐5	2.1	2.0	2.2	2.1	2.0	2.3	2.4	2.2	2.6
Greens & beans	0‐5	1.6	1.3	1.9	1.5	1.3	1.8	1.6	1.3	1.9
Whole grains	0‐10	3.4	3.0	3.8	2.7	2.4	2.9	2.4	2.1	2.6
Dairy	0‐10	10.0	10.0	10.0	9.0	8.6	9.3	8.0	7.5	8.5
Total protein foods	0‐5	4.0	3.8	4.2	4.2	4.0	4.3	4.9	4.6	5.0
Seafood & plant proteins	0‐5	2.9	2.4	3.5	2.7	2.4	3.0	3.4	2.8	3.9
Fatty acids	0‐10	2.3	1.9	2.7	2.9	2.6	3.2	3.6	3.1	4.1
Refined grains	0‐10	5.9	5.5	6.4	4.4	4.0	4.7	4.5	4.1	4.8
Sodium	0‐10	5.8	5.5	6.2	5.0	4.7	5.3	4.1	3.7	4.4
Added sugars	0‐10	6.8	6.6	7.1	5.4	5.1	5.7	5.1	4.7	5.4
Saturated fats	0‐10	5.3	4.9	5.8	5.5	5.2	5.8	5.8	5.4	6.1
Total score	0‐100	60.2	58.5	62.1	54.0	52.5	55.4	52.0	50.3	53.7
		Overweight (85th ≤ BMI < 95th Percentile)								
		2‐5 y (n = 317)			6‐11 y (n = 540)			12‐18 y (n = 539)		
Component	Range	Mean	95% CI		Mean	95% CI		Mean	95% CI	
Total fruits	0‐5	5.0	4.9	5.0	3.7	3.2	4.3	2.8	2.3	3.4
Whole fruits	0‐5	5.0	5.0	5.0	4.9	4.1	5.0	3.6	2.6	4.6
Total vegetables	0‐5	2.1	1.8	2.4	2.2	2.0	2.4	2.5	2.2	2.8
Greens & beans	0‐5	1.3	1.0	1.6	2.0	1.4	2.5	1.7	1.2	2.1
Whole grains	0‐10	2.9	2.4	3.5	2.5	2.1	2.9	2.2	1.7	2.6
Dairy	0‐10	10.0	10.0	10.0	8.7	8.1	9.3	7.4	6.7	8.1
Total protein foods	0‐5	4.0	3.6	4.3	4.5	4.2	4.9	5.0	4.7	5.0
Seafood & plant proteins	0‐5	3.0	2.3	3.6	3.4	2.6	4.2	3.0	2.3	3.7
Fatty acids	0‐10	2.2	1.4	3.1	3.1	2.4	3.8	4.0	3.4	4.5
Refined grains	0‐10	6.3	5.7	7.0	4.2	3.4	5.0	4.9	4.2	5.6
Sodium	0‐10	5.6	5.2	6.1	4.8	4.0	5.5	3.7	3.0	4.4
Added sugars	0‐10	7.0	6.6	7.5	5.8	5.3	6.2	5.1	4.4	5.8
Saturated fats	0‐10	4.8	3.9	5.7	5.2	4.3	6.1	5.8	5.4	6.1
Total score	0‐100	59.2	56.4	62.1	54.9	52.1	57.5	51.5	49.4	53.7
		Obese (BMI ≥ 95th Percentile)								
		2‐5 y (n = 272)			6‐11 y (n = 657)			12‐18 y (n = 674)		
Component	Range	Mean	95% CI		Mean	95% CI		Mean	95% CI	
Total fruits	0‐5	4.9	4.3	5.0	3.5	3.1	3.9	2.8	2.2	3.3
Whole fruits	0‐5	5.0	4.9	5.0	4.5	3.9	5.0	3.5	2.6	4.3
Total vegetables	0‐5	2.2	1.9	2.4	2.3	2.1	2.4	2.8	2.3	3.2
Greens & beans	0‐5	1.5	0.8	2.2	1.5	1.2	1.9	2.2	0.9	3.6
Whole grains	0‐10	2.7	2.1	3.3	2.5	2.1	2.8	2.0	1.6	2.4
Dairy	0‐10	10.0	9.5	10.0	9.0	8.3	9.6	8.0	7.3	8.7
Total protein foods	0‐5	4.6	4.2	4.9	4.7	4.3	5.0	5.0	5.0	5.0
Seafood & plant proteins	0‐5	2.5	1.9	3.2	3.1	2.5	3.8	2.8	1.9	3.8
Fatty acids	0‐10	2.9	2.3	3.6	2.9	2.4	3.5	4.2	3.6	4.9
Refined grains	0‐10	6.4	5.7	7.0	4.0	3.4	4.6	4.8	4.1	5.6
Sodium	0‐10	5.1	4.3	5.9	4.3	3.9	4.7	3.3	2.6	4.1
Added sugars	0‐10	6.1	5.5	6.8	6.1	5.6	6.5	4.9	4.1	5.7
Saturated fats	0‐10	5.9	5.1	6.6	5.1	4.6	5.6	6.3	5.8	6.9
Total score	0‐100	59.7	57.2	62.2	53.3	50.8	55.8	52.6	48.6	56.9

*Note.* HEI scores are calculated using population ratio method.

Abbreviations: BMI, body mass index (kg/m^2^); CI, confidence interval; HEI, Healthy Eating Index; NHANES, National Health and Nutrition Examination Survey.

**Table 4:** HEI‐2015 scores for children 2‐18 years of age by BMI category and gender: NHANES 2009‐2014 (N = 8894)

		Underweight (BMI < 5th Percentile)						Normal Weight (5th ≤ BMI < 85th Percentile)					
		Boys (n = 164)			Girls (n = 141)			Boys (n = 2836)			Girls (n = 2754)		
Component	Range	Mean	95% CI		Mean	95% CI		Mean	95% CI		Mean	95% CI	
Total fruits	0‐5	2.8	2.3	3.4	3.4	2.5	4.4	3.7	3.4	4.0	3.8	3.5	4.1
Whole fruits	0‐5	3.8	2.9	4.7	3.8	2.8	4.9	4.5	4.2	4.9	4.9	4.4	5.0
Total vegetables	0‐5	2.0	1.5	2.4	2.0	1.6	2.5	2.1	2.0	2.3	2.4	2.3	2.5
Greens & beans	0‐5	1.4	0.1	2.7	1.7	0.8	2.7	1.5	1.2	1.7	1.7	1.6	1.9
Whole grains	0‐10	2.1	1.7	2.5	2.7	1.7	3.7	2.7	2.5	2.9	2.6	2.4	2.9
Dairy	0‐10	9.0	7.7	10.0	9.7	8.5	10.0	9.2	8.8	9.6	8.8	8.3	9.2
Total protein foods	0‐5	4.2	3.6	4.8	4.4	3.8	5.0	4.7	4.4	4.9	4.3	4.0	4.5
Seafood & plant proteins	0‐5	2.6	1.6	3.5	3.1	1.9	4.3	2.9	2.5	3.4	3.1	2.8	3.5
Fatty acids	0‐10	1.7	0.7	2.7	2.1	0.9	3.5	3.0	2.6	3.4	3.2	2.9	3.4
Refined grains	0‐10	4.2	3.1	5.3	4.5	2.6	6.3	4.7	4.4	5.0	4.7	4.4	5.1
Sodium	0‐10	5.3	4.5	6.0	4.3	2.8	5.8	4.6	4.2	5.0	4.9	4.6	5.2
Added sugars	0‐10	5.3	4.2	6.5	6.3	5.3	7.3	5.6	5.4	5.9	5.4	5.1	5.8
Saturated fats	0‐10	4.0	2.9	5.1	4.4	3.2	5.8	5.7	5.4	5.9	5.5	5.3	5.8
Total score	0‐100	48.2	43.9	52.7	52.7	47.1	58.2	55.0	53.6	56.4	55.3	54.0	56.6
		Overweight (85th ≤ BMI < 95th Percentile)						Obese (≥95th Percentile)					
		Boys (n = 701)			Girls (n = 695)			Boys (n = 837)			Girls (n = 766)		
Component	Range	Mean	95% CI		Mean	95% CI		Mean	95% CI		Mean	95% CI	
Total fruits	0‐5	3.7	3.2	4.3	3.6	3.1	4.1	3.3	2.8	3.8	3.4	3.1	3.7
Whole fruits	0‐5	4.6	3.7	5.0	4.5	3.8	5.0	4.1	3.3	5.0	4.2	3.8	4.7
Total vegetables	0‐5	2.3	2.0	2.5	2.4	2.1	2.6	2.4	2.2	2.7	2.6	2.2	3.0
Greens & beans	0‐5	1.7	1.1	2.2	1.8	1.3	2.2	1.9	1.2	2.5	1.9	1.0	2.7
Whole grains	0‐10	2.3	1.9	2.7	2.6	2.2	2.9	2.2	1.9	2.6	2.3	1.8	2.8
Dairy	0‐10	8.5	8.0	8.9	8.9	8.3	9.6	8.9	8.2	9.6	8.2	7.8	8.7
Total protein foods	0‐5	4.9	4.4	5.0	4.7	4.3	5.0	5.0	4.8	5.0	4.8	4.5	5.0
Seafood & plant proteins	0‐5	3.0	2.5	3.4	3.4	2.6	4.1	3.1	2.5	3.8	2.7	2.2	3.1
Fatty acids	0‐10	3.2	2.7	3.8	3.5	3.0	4.0	3.3	2.8	3.9	3.9	3.4	4.4
Refined grains	0‐10	4.7	4.1	5.4	5.1	4.5	5.6	5.0	4.4	5.6	4.2	3.6	4.8
Sodium	0‐10	4.4	3.8	5.0	4.5	4.0	5.1	4.1	3.5	4.7	3.7	3.1	4.2
Added sugars	0‐10	5.6	5.1	6.2	5.8	5.3	6.2	5.3	4.7	5.9	5.7	5.4	6.1
Saturated fats	0‐10	5.4	4.7	6.0	5.4	4.9	5.9	5.8	5.2	6.3	5.8	5.3	6.4
Total score	0‐100	54.2	51.7	56.5	56.0	53.5	58.6	54.4	51.7	57.2	53.4	50.4	56.5

*Note.* HEI scores are calculated using population ratio method.

Abbreviations: BMI, body mass index (kg/m^2^); CI, confidence interval; HEI, Healthy Eating Index; NHANES, National Health and Nutrition Examination Survey.

**Table 5:** HEI‐2015 scores for children 2‐18 years of age by BMI category and race/ethnicity: NHANES 2009‐2014 (N = 8894)

	Normal Weight														
	NHB (n = 1342)			NHW (n = 1670)			MA (n = 1179)			OH (n = 583)			OR (n = 816)		
Component	Mean	95% CI		Mean	95% CI		Mean	95% CI		Mean	95% CI		Mean	95% CI	
Total fruits	3.5	3.2	3.7	3.6	3.4	3.8	4.3	3.9	4.8	4.1	3.5	4.6	4.1	3.6	4.6
Whole fruits	3.5	3.2	3.9	4.8	4.4	5.0	5.0	4.7	5.0	4.5	3.5	5.0	4.7	4.2	5.0
Total vegetables	2.1	2.0	2.3	2.2	2.1	2.4	2.5	2.3	2.6	2.3	2.1	2.5	2.4	2.1	2.7
Greens & beans	1.5	1.2	1.8	1.4	1.1	1.6	2.2	1.7	2.7	2.0	1.5	2.5	2.0	1.5	2.5
Whole grains	2.4	2.1	2.7	2.8	2.5	3.1	2.2	1.9	2.4	2.8	2.2	3.4	2.9	2.5	3.4
Dairy	7.1	6.6	7.6	9.6	9.1	10.0	9.1	8.7	9.6	8.2	7.8	8.6	8.6	8.0	9.2
Total protein foods	4.8	4.6	5.0	4.2	3.9	4.5	4.8	4.5	5.0	4.8	4.3	5.0	4.8	4.4	5.0
Seafood & plant proteins	2.4	2.1	2.8	2.9	2.5	3.4	3.6	2.9	4.3	3.2	2.3	4.1	3.6	2.9	4.3
Fatty acids	4.4	4.0	4.7	2.7	2.3	3.1	3.1	2.7	3.5	3.4	2.9	3.9	3.2	2.8	3.6
Refined grains	5.1	4.7	5.4	5.0	4.7	5.4	3.5	3.1	4.0	4.9	4.1	5.6	3.8	2.8	4.8
Sodium	4.8	4.5	5.1	4.9	4.5	5.3	4.8	4.4	5.1	5.0	4.6	5.4	3.2	2.3	4.1
Added sugars	5.4	5.1	5.7	5.0	4.7	5.4	6.6	6.3	6.9	5.8	5.3	6.2	7.3	6.6	7.9
Saturated fats	5.9	5.7	6.2	5.4	5.1	5.7	5.4	5.1	5.8	6.2	5.7	6.7	6.2	5.8	6.7
Total score	53.0	51.5	54.5	54.6	53.3	55.9	57.1	55.0	59.2	57.0	53.9	60.0	56.8	54.2	59.4
	Overweight														
	NHB (n = 341)			NHW (n = 344)			MA (n = 384)			OH (n = 178)			OR (n = 149)		
Component	Mean	95% CI		Mean	95% CI		Mean	95% CI		Mean	95% CI		Mean	95% CI	
Total fruits	3.0	2.6	3.4	3.6	2.9	4.3	4.3	3.7	4.9	3.4	2.7	4.2	4.4	3.3	5.0
Whole fruits	2.6	2.1	3.0	4.6	3.4	5.0	5.0	4.7	5.0	4.1	2.9	5.0	5.0	4.4	5.0
Total vegetables	2.2	2.0	2.5	2.1	1.9	2.4	2.7	2.3	3.0	2.2	1.9	2.4	2.8	2.3	3.2
Greens & beans	1.4	0.8	1.9	1.3	0.9	1.8	2.6	2.0	3.2	1.8	0.9	2.7	2.9	1.3	4.3
Whole grains	2.2	1.7	2.7	2.5	2.0	3.0	2.1	1.7	2.6	2.2	1.4	3.0	3.2	2.1	4.2
Dairy	7.3	6.4	8.1	9.1	8.4	9.7	8.6	8.0	9.3	8.5	7.5	9.6	9.0	7.9	10.0
Total protein foods	4.7	4.2	5.0	4.8	4.2	5.0	4.9	4.5	5.0	4.6	4.1	5.0	4.7	3.9	5.0
Seafood & plant proteins	2.3	1.4	3.2	2.8	2.3	3.4	3.6	2.8	4.4	3.2	2.2	4.1	4.5	2.2	5.0
Fatty acids	4.6	3.9	5.2	2.8	2.2	3.4	3.9	3.3	4.6	3.0	2.1	4.1	3.6	2.3	5.1
Refined grains	4.7	3.6	5.8	5.4	4.6	6.2	3.9	3.3	4.6	4.3	3.1	5.5	4.8	3.7	6.0
Sodium	4.2	3.4	5.0	4.6	3.8	5.3	4.6	3.9	5.2	5.3	4.4	6.1	3.1	1.8	4.4
Added sugars	5.2	4.5	6.0	5.5	4.9	6.1	6.5	6.1	6.9	4.9	4.1	5.7	7.0	6.1	7.9
Saturated fats	6.0	5.4	6.6	4.8	4.1	5.5	6.2	5.7	6.8	5.9	5.1	6.6	5.6	4.0	7.3
Total score	50.3	47.1	53.6	53.9	50.9	56.6	59.0	55.7	62.2	53.3	50.0	56.6	60.4	55.0	65.4
	Obese														
	NHB (n = 438)			NHW (n = 359)			MA (n = 458)			OH (n = 203)			OR (n = 145)		
Component	Mean	95% CI		Mean	95% CI		Mean	95% CI		Mean	95% CI		Mean	95% CI	
Total fruits	3.3	2.7	3.9	3.1	2.5	3.7	3.7	3.2	4.2	3.9	3.1	4.7	3.1	2.1	4.0
Whole fruits	3.6	2.9	4.4	4.4	3.5	5.0	4.2	3.5	4.9	4.5	3.4	5.0	3.7	2.4	4.8
Total vegetables	2.2	2.0	2.5	2.5	2.0	3.1	2.5	2.3	2.7	2.6	2.1	3.0	2.6	1.9	3.2
Greens & beans	1.6	1.1	2.1	1.7	0.5	3.1	2.0	1.6	2.5	2.4	1.5	3.3	2.2	0.8	3.7
Whole grains	2.3	1.9	2.8	2.3	1.9	2.7	1.9	1.6	2.2	2.6	1.7	3.5	2.4	1.5	3.3
Dairy	7.0	6.3	7.8	9.1	8.4	9.8	8.6	7.7	9.6	8.6	7.8	9.5	9.1	7.7	10.0
Total protein foods	5.0	4.9	5.0	4.8	4.5	5.0	5.0	4.6	5.0	5.0	4.6	5.0	4.7	4.1	5.0
Seafood & plant proteins	2.7	2.0	3.3	2.7	2.0	3.5	3.2	2.5	3.9	3.6	2.6	4.6	3.2	1.8	4.7
Fatty acids	4.2	3.8	4.7	3.4	2.7	4.2	3.7	2.9	4.4	3.6	2.4	4.8	2.9	1.8	3.9
Refined grains	5.3	4.5	6.0	4.9	4.2	5.7	3.7	2.9	4.4	4.6	3.5	5.8	4.4	3.4	5.3
Sodium	4.4	3.8	4.9	3.8	3.0	4.5	4.0	3.3	4.6	4.3	3.6	5.0	3.2	1.6	4.9
Added sugars	5.2	4.7	5.8	4.7	4.0	5.5	6.5	5.9	7.0	6.6	5.8	7.3	6.6	5.8	7.5
Saturated fats	5.7	5.2	6.3	6.0	5.3	6.6	5.6	5.1	6.2	5.9	4.9	6.8	5.0	4.0	6.0
Total score	52.6	49.9	55.5	53.5	49.3	57.9	54.5	51.8	57.4	58.0	53.0	63.1	53.0	49.3	56.8

*Note.* HEI scores are calculated using population ratio method. Underweight racial/ethnic groups are not reported due to small samples sizes resulting in unreliable estimates.

Abbreviations: BMI, body mass index (kg/m^2^); CI, confidence interval; HEI, Healthy Eating Index; NHANES, National Health and Nutrition Examination Survey; NHB, non‐Hispanic black; NHW, non‐Hispanic white; MA, Mexican American; OH, other Hispanic; OR, other race.

**Table 6:** HEI‐2015 sores for children 2‐18 years of age by BMI category and PIR class: NHANES 2009‐2014 (N = 8894)

		Underweight (BMI < 5th Percentile)						Normal Weight (5th ≤ BMI < 85th Percentile)					
		PIR < 1 (n = 91)			PIR ≥ 1 (n = 191)			PIR < 1 (n = 1725)			PIR ≥ 1 (n = 3418)		
Component	Range	Mean	95% CI		Mean	95% CI		Mean	95% CI		Mean	95% CI	
Total fruits	0‐5	2.6	1.8	3.5	3.2	2.6	3.8	3.6	3.3	3.8	3.8	3.6	4.1
Whole fruits	0‐5	3.0	1.8	4.3	3.8	3.1	4.6	4.1	3.7	4.5	4.9	4.5	5.0
Total vegetables	0‐5	2.2	1.7	2.7	1.9	1.5	2.3	2.3	2.2	2.5	2.2	2.1	2.4
Greens & beans	0‐5	1.1	0.2	2.0	1.7	0.6	2.8	1.9	1.5	2.3	1.5	1.4	1.7
Whole grains	0‐10	2.0	1.5	2.6	2.5	1.9	3.2	2.4	2.1	2.6	2.8	2.5	3.0
Dairy	0‐10	8.9	7.1	10.0	9.6	8.3	10.0	8.6	8.2	9.1	9.1	8.7	9.5
Total protein foods	0‐5	4.4	3.7	5.0	4.3	3.8	4.9	4.7	4.4	4.9	4.4	4.2	4.7
Seafood & plant proteins	0‐5	2.9	1.3	4.6	2.8	1.9	3.8	2.8	2.4	3.3	3.0	2.6	3.4
Fatty acids	0‐10	2.0	1.0	3.1	1.9	0.7	3.2	3.2	2.8	3.6	3.1	2.7	3.4
Refined grains	0‐10	3.8	2.4	5.2	4.6	3.5	5.8	4.6	4.2	4.9	4.9	4.6	5.1
Sodium	0‐10	4.9	3.8	6.0	4.7	3.7	5.7	4.5	4.2	4.8	4.8	4.4	5.1
Added sugars	0‐10	4.9	3.5	6.4	6.0	5.1	6.8	5.8	5.4	6.1	5.4	5.1	5.7
Saturated fats	0‐10	4.8	3.7	6.0	4.0	2.7	5.3	5.4	5.1	5.8	5.7	5.4	5.9
Total score	0‐100	47.7	42.8	52.8	51.1	46.6	55.8	53.9	52.2	55.6	55.6	54.5	56.6
		Overweight (85th ≤ BMI < 95th Percentile)						Obese (BMI ≥ 95th Percentile)					
		PIR < 1 (n = 429)			PIR ≥ 1 (n = 834)			PIR < 1 (n = 552)			PIR ≥ 1 (n = 906)		
Component	Range	Mean	95% CI		Mean	95% CI		Mean	95% CI		Mean	95% CI	
Total fruits	0‐5	3.4	3.0	3.9	3.7	3.1	4.3	3.2	2.8	3.5	3.4	2.9	3.9
Whole fruits	0‐5	4.0	3.3	4.8	4.6	3.7	5.0	4.0	3.5	4.6	4.3	3.5	5.0
Total vegetables	0‐5	2.4	2.2	2.7	2.2	2.1	2.4	2.5	2.2	2.8	2.5	2.1	2.9
Greens & beans	0‐5	2.4	1.6	3.2	1.4	1.1	1.8	1.7	1.3	2.1	2.0	1.1	2.9
Whole grains	0‐10	2.3	1.8	2.7	2.6	2.2	3.0	2.0	1.6	2.5	2.3	2.0	2.6
Dairy	0‐10	8.4	7.6	9.3	8.7	8.2	9.3	8.8	8.2	9.5	8.6	8.0	9.2
Total protein foods	0‐5	4.9	4.2	5.0	4.7	4.3	5.0	5.0	4.8	5.0	4.9	4.7	5.0
Seafood & plant proteins	0‐5	3.1	2.1	4.0	3.0	2.5	3.6	2.5	2.0	3.0	3.1	2.5	3.7
Fatty acids	0‐10	3.7	3.0	4.5	3.1	2.6	3.7	3.7	3.1	4.2	3.5	2.9	4.1
Refined grains	0‐10	5.1	4.3	5.8	4.8	4.2	5.5	4.6	4.1	5.1	4.8	4.1	5.4
Sodium	0‐10	3.9	3.2	4.7	4.6	4.1	5.1	3.8	2.9	4.7	4.0	3.5	4.5
Added sugars	0‐10	5.7	5.0	6.4	5.7	5.3	6.2	6.1	5.4	6.6	5.2	4.7	5.8
Saturated fats	0‐10	5.8	5.2	6.5	5.2	4.5	5.8	5.4	5.0	5.9	5.8	5.3	6.4
Total score	0‐100	55.2	52.5	57.9	54.4	52.0	56.7	53.3	51.1	55.5	54.3	51.2	57.6

*Note.* HEI scores are calculated using population ratio method.

Abbreviations: BMI, body mass index (kg/m^2^); CI, confidence interval; HEI, Healthy Eating Index; NHANES, National Health and Nutrition Examination Survey; PIR, poverty to income ratio.

A significant difference was present for BMI category within gender for boys (Table 4). The mean total score was significantly higher for boys with normal weight vs underweight (55.0 vs 48.2). Significant differences were present for race/ethnicity within BMI category (Table 5 and Figure 3 [normal weight and overweight only]). For children with normal weight, the mean total score was significantly higher for Mexican Americans versus non‐Hispanic blacks (57.1 vs 53.0). For children with overweight, mean total scores were significantly higher for Mexican Americans and other races vs non‐Hispanic blacks (59.0 and 60.4 vs 50.3). Significant differences were not found for PIR class within BMI category or BMI category within PIR class (Table 6).

### 3.2 HEI‐2015 component scores

**Figure 4:**
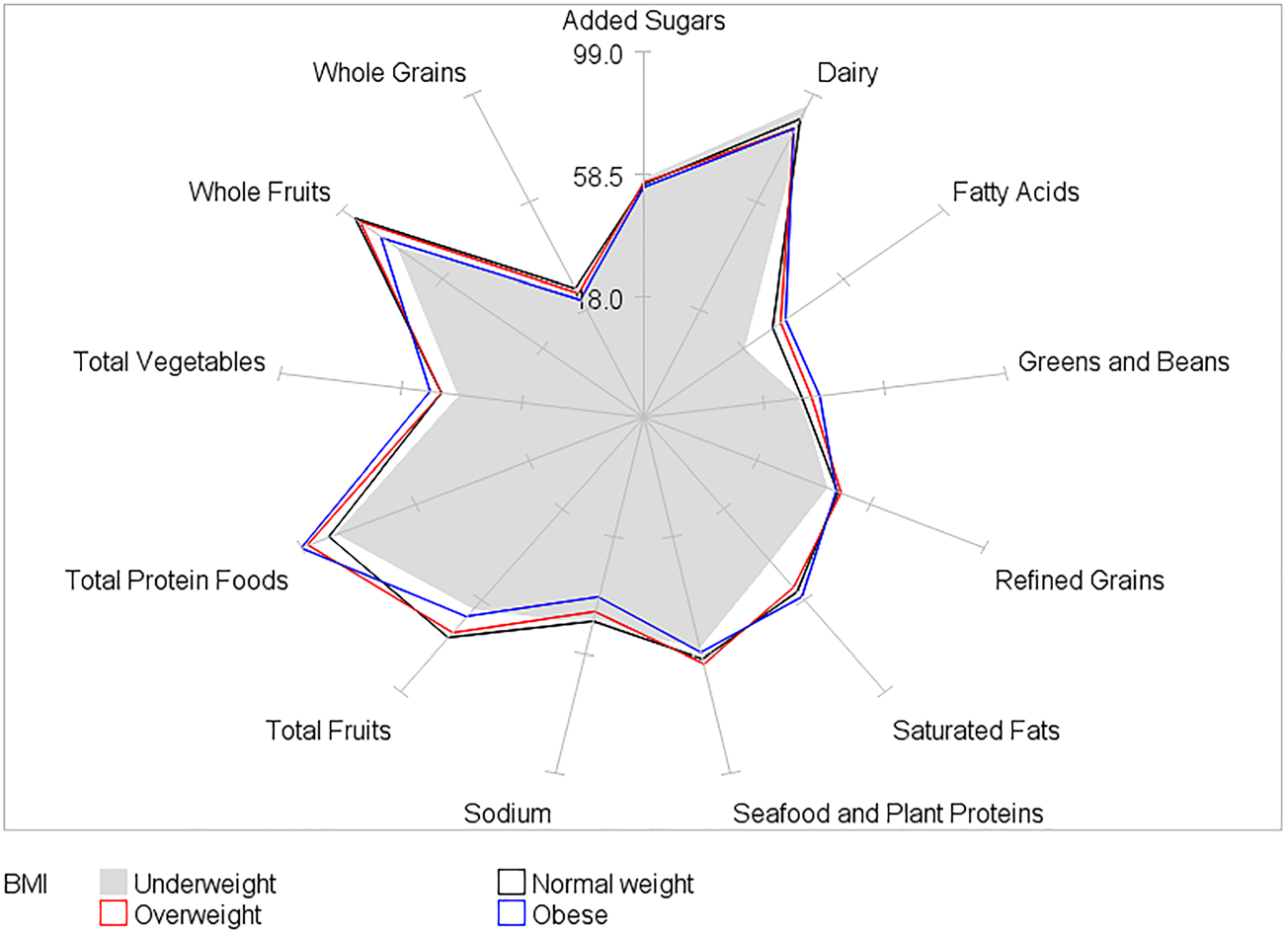
Ratios of HEI‐2015 mean component scores to maximum scores for children 2‐18 years of age by body mass index category: NHANES 2009‐2014.HEI scores are calculated using population ratio method. Spokes represent percent of maximum score. Body mass index (BMI) categories: underweight BMI < 5th percentile; normal weight 5th ≤ BMI < 85th percentile; overweight 85th ≤ BMI < 95th percentile; obese BMI ≥ 95th percentile. HEI, Healthy Eating Index; NHANES, National Health and Nutrition Examination Survey

**Figure 5:**
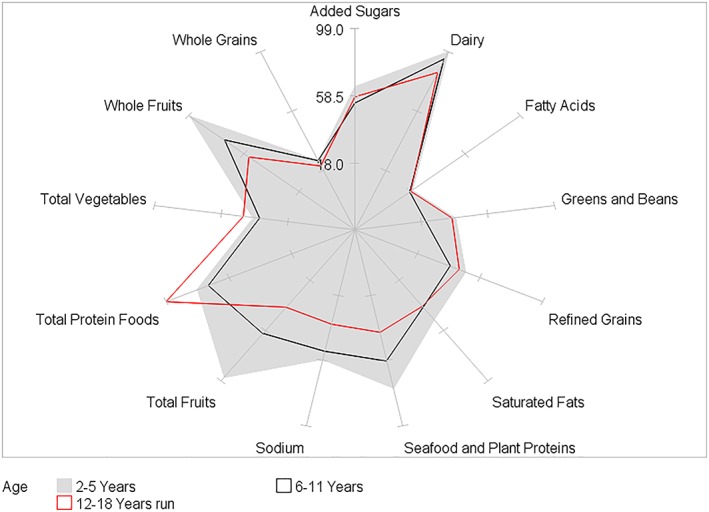
Ratios of HEI‐2015 mean component scores to maximum scores for children 2‐18 years of age with underweight by age group: NHANES 2009‐2014. HEI scores are calculated using population ratio method. Spokes represent percent of maximum score. Underweight BMI < 5th percentile. HEI, Healthy Eating Index; NHANES, National Health and Nutrition Examination Survey

**Figure 6:**
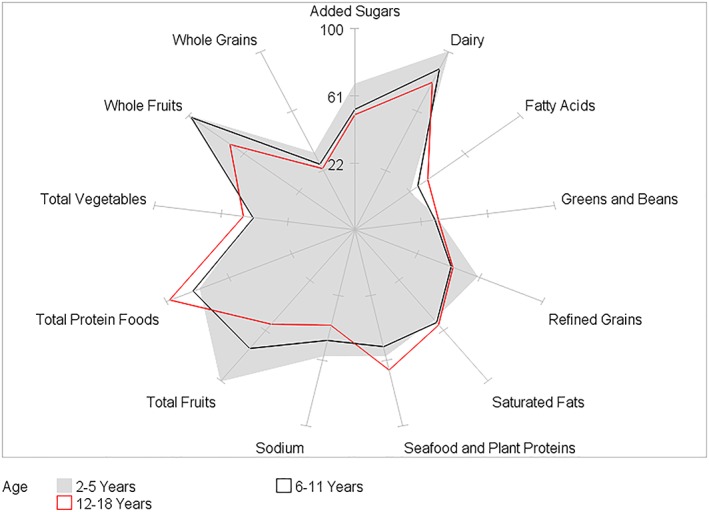
Ratios of HEI‐2015 mean component scores to maximum scores for children 2‐18 years of age with normal weight by age group: NHANES 2009‐2014. HEI scores are calculated using population ratio method. Spokes represent percent of maximum score. Normal weight 5th ≤ BMI < 85th percentile. HEI, Healthy Eating Index; NHANES, National Health and Nutrition Examination Survey

**Figure 7:**
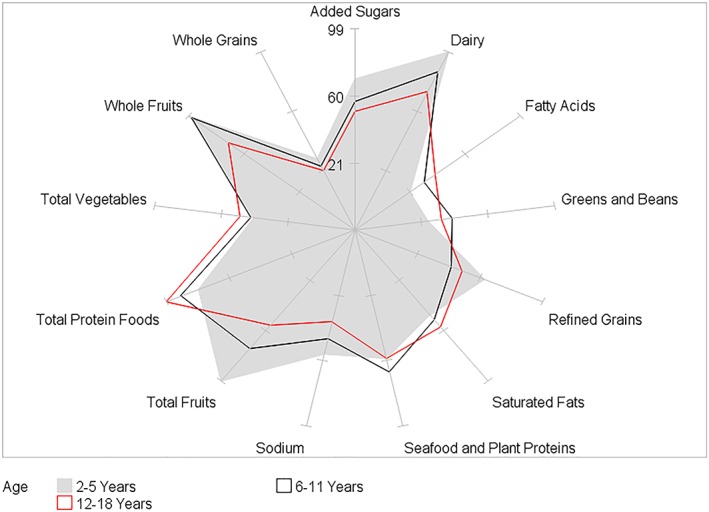
Ratios of HEI‐2015 mean component scores to maximum scores for children 2‐18 years of age with overweight by age group: NHANES 2009‐2014. HEI scores are calculated using population ratio method. Spokes represent percent of maximum score. Overweight 85th ≤ BMI < 95th percentile. HEI, Healthy Eating Index; NHANES, National Health and Nutrition Examination Survey

**Figure 8:**
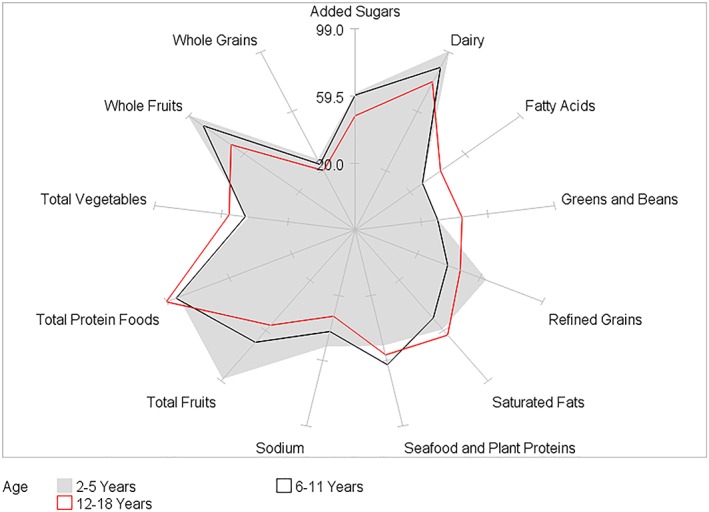
Ratios of HEI‐2015 mean component scores to maximum scores for children 2‐18 years of age with obesity by age group: NHANES 2009‐2014. HEI scores are calculated using population ratio method. Spokes represent percent of maximum score. Obese BMI ≥ 95th percentile. HEI, Healthy Eating Index; NHANES, National Health and Nutrition Examination Survey

**Figure 9:**
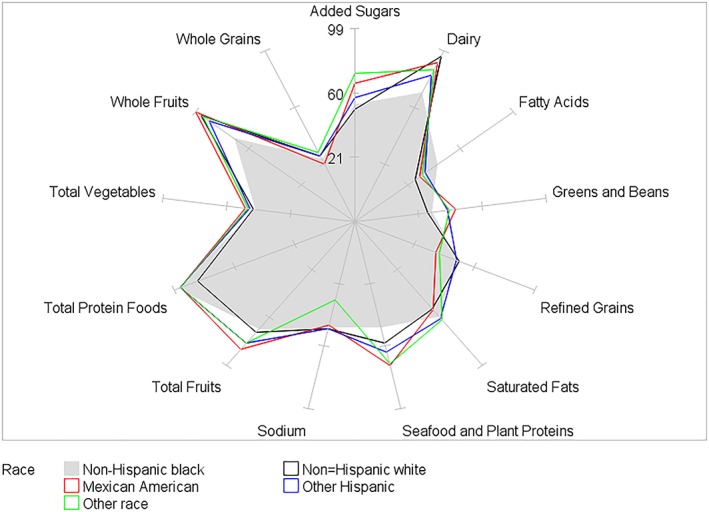
Ratios of HEI‐2015 mean component scores to maximum scores for children 2‐18 years of age with normal weight by race/ethnicity: NHANES 2009‐2014. HEI scores are calculated using population ratio method. Spokes represent percent of maximum score. Normal weight 5th ≤ BMI < 85th percentile. HEI, Healthy Eating Index; NHANES, National Health and Nutrition Examination Survey

**Figure 10:**
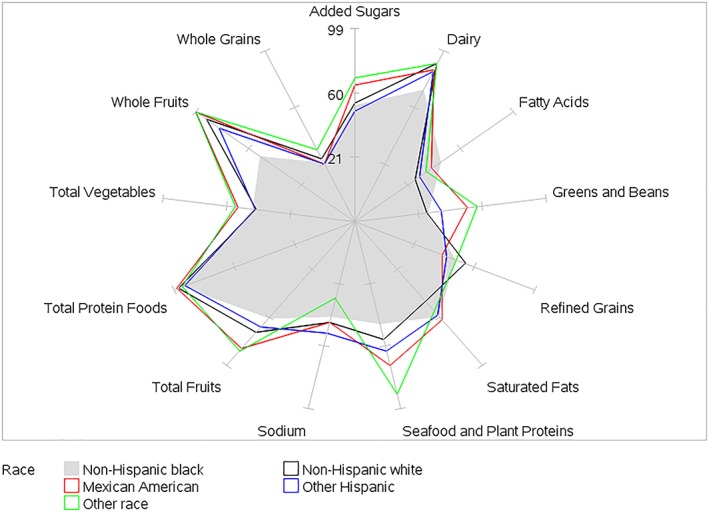
Ratios of HEI‐2015 mean component scores to maximum scores for children 2‐18 years of age with overweight by race/ethnicity: NHANES 2009‐2014. HEI scores are calculated using population ratio method. Spokes represent percent of maximum score. Overweight 85th ≤ BMI < 95th percentile. HEI, Healthy Eating Index; NHANES, National Health and Nutrition Examination Survey

HEI‐2015 mean component scores for children by BMI category and by age, gender, race/ethnicity, and PIR within BMI category are presented in Tables 2, 3, 4, 5, 6 and Figures 4, 5, 6, 7, 8, 9, 10 (Figures 5, 6, 7, 8 and 10). Among BMI categories (Table 2 and Figure 4), the mean total protein foods score was significantly higher for children with obesity versus children with underweight and normal weight. The mean fatty acids score was significantly higher for children with overweight and obesity versus children with underweight. The mean sodium score was significantly higher for children with normal weight versus children with obesity. The mean saturated fat scores were significantly higher for children with normal weight and obesity versus children with underweight.

Age group differences were somewhat similar among BMI categories (Table 3 and Figures 5, 6, 7, 8). In all four BMI categories, the mean total and whole fruits scores were higher for the youngest versus one or both of the older age groups, while the total protein foods score was higher for the oldest versus one or both of the younger age groups. For children with normal weight, overweight, and obesity, the dairy, refined grains, and sodium scores were higher in the youngest versus one or both of the older age groups, while the fatty acid scores were higher in the oldest versus one of the younger age groups. For children with normal weight and overweight, the added sugars score was higher in the youngest versus the two older age groups. Additionally, for the oldest age group, the fatty acid and saturated fat mean scores were higher for children with normal, overweight, and obesity versus children with underweight.

For gender (Table 4), the mean fatty acids score was significantly higher for boys with obesity versus boys with underweight. Similarly, the mean saturated fats scores were significantly higher for boys with normal weight and obesity versus boys with underweight. The mean sodium score was significantly higher for girls with normal weight versus girls with obesity.

For race/ethnicity (Table 5, Figure 9 [normal weight], and Figure 10 [overweight]), the mean whole fruits score was significantly higher for non‐Hispanic black children with normal weight versus those with overweight, while the mean added sugars score was significantly higher for other Hispanic children with obesity versus those with overweight. For children with normal weight, racial/ethnic differences in mean scores were found for all components except for total vegetables. For children with overweight, racial/ethnic differences in mean scores were found for seven components—total fruits, whole fruits, greens and beans, dairy, fatty acids, added sugars, and saturated fats. For children with obesity, racial/ethnic differences were found for two components—refined grains and added sugars. For PIR ≥ 1 (Table 6), the mean saturated fats score was significantly higher for children with normal weight versus children with underweight.

## 4 DISCUSSION

The study represents the most up‐to‐date estimates of diet quality for nationally representative populations of noninstitutionalized children in the United States according to categories of BMI and sociodemographic characteristics within BMI categories. Significant differences in HEI‐2015 mean total scores were not observed among BMI categories. However, lower component scores were observed for total protein foods, fatty acids, and saturated fats in children with underweight, and lower sodium scores were observed in children with obesity. Results from two studies investigating the relationship between intake of fatty acids and task performance in children indicated that neither HEI‐2010 scores nor n‐6/n‐3 fatty acid ratios were associated with BMI 16. However, the studies were conducted in the Southeast and therefore are not nationally representative 16. Likewise, a systematic review and meta‐analysis found no relationship between reduced saturated fats intake and BMI in children 17. Similar to the present study, a positive relationship between diet quality related to sodium intake and body weight in children has been reported in two other studies 18, 19.

Significant and similar age group differences in total diet quality and the majority of diet quality components were found in children with normal weight, overweight, and obesity. Age group differences also were similar in children with underweight, but significance was not reached likely due to smaller samples sizes. In agreement with the present study, others also have reported negative relationships between child age and intakes of fruit, whole and refined grains, and dairy (less healthful as age increases) 20, 21, 22, positive relationships between age and intakes of protein foods and fatty acids (more healthful as age increases) 20, and positive relationships between age and intakes of sodium and added sugars (less healthful as age increases) 18, 20, 23. The present study's results also confirm the lack of significant relationships between age and vegetable intake reported by some researchers 21.

Significant racial/ethnic differences in total diet quality and most components of diet quality were found, notably in children with normal weight. Others also have reported racial/ethnic differences in diet quality among children, although not within BMI categories. Similar to the present study, HEI‐2005 and HEI‐2010 total scores were highest for Mexican American children and lowest for non‐Hispanic black children 24, 25. Contrary to the present study, racial/ethnic differences were found for HEI‐2005 component scores for total vegetables but not for oils, saturated fats, or discretionary calories 24. Comparisons should be interpreted cautiously due to different versions of HEI used, number of race/ethnicity categories reported, and analysis for race/ethnicity without regard to BMI category versus race/ethnicity within BMI categories.

In the present study, mean total diet quality scores were less than 60 for the populations of children categorized by BMI and sociodemographic characteristics within BMI categories with two exceptions—children 2 to 5 years of age with normal weight (score = 60.2) and children of other race with overweight (score = 60.4). Scores less than 60 correspond to a qualitative failing (“F”) grade while scores less than 70 correspond to a grade of D 9. Thus, although differences were apparent among some populations of children in the United States, all children's diets are poor and in need of improvement, regardless of BMI status, age, gender, race/ethnicity, and PIR class. In particular, mean component scores were generally low (<50% of maximum score) for greens and beans, whole grains, fatty acids, and sodium for most populations of children included in the study. Conversely, mean component scores were generally higher (>50% of maximum score) for total and whole fruits, dairy, and total protein foods. Focusing on components of the diet most in need of improvement will likely have a large effect on overall diet quality than components that are approaching their maximum score.

In a systematic review of interventions to prevent global childhood overweight and obesity, the authors concluded that their findings support recommendations that schools should be the focal point of child obesity prevention efforts, and school‐based interventions should include diet, physical activity, and home components 26. The home component may be particularly relevant for younger children because parents report feeling more responsibility for feeding their younger children than their older children 27. In black children, the potential for school and child care interventions to effectively improve nutrition and physical activity was found in three reviews 28. In another systematic review of family‐based and institutional nutrition interventions, the authors concluded that the most effective strategies for improving child eating habits included the use of rewards, cartoon characters promoting healthy foods, modelling by teachers, and the use of older peer educators 29. Cultural tailoring of nutrition interventions may be particularly relevant for Hispanic child populations because food choices are affected by culturally mediated health beliefs and by popular food classifications (eg, hot‐cold) 30.

The use of nationally representative child datasets, the most recent HEI, and the population ratio method for estimating diet quality scores are strengths of the study. The population ratio method provides reasonably accurate population level estimates of usual intake means 31; however, it does not adjust for skewness or account for correlations between dietary constituents and energy intake. Limitations of this study include the use of self‐reported dietary intake, which can result in biased measurement (eg, under‐reporting intake) and the large number of comparisons performed, which can result in inflated type 1 error rates. However, reporting estimates of diet quality for various nationally representative child populations by categories of BMI was the focus of the study (not statistical comparisons).

In conclusion, results from our analysis of nationally representative data suggests that commonly examined subpopulations of children, defined by weight status and sociodemographic characteristics, are eating very poorly. For all populations of children represented in this study, total diet quality scores were low both quantitatively and qualitatively. Total and most components of diet quality did not significantly differ among child populations classified by BMI status. Within BMI categories, the majority of significant diet quality differences were found for age and race/ethnicity groups. These results suggest that much work still needs to be accomplished to improve the diet quality of children in the United States, regardless of BMI status. Researchers, practitioners, and clinicians can use these estimates when designing nutrition interventions, setting nutrition policies, and counselling paediatric patients and their caregivers about the importance of a healthful diet. Stakeholders may need to address or target specific dietary components with low quality in various child populations to have the greatest effect on improving nutrition nationwide.

## CONFLICT OF INTEREST STATEMENT

No conflict of interest was declared.
